# Sleep disorders and addiction A study of 100 patients

**DOI:** 10.1192/j.eurpsy.2024.633

**Published:** 2024-08-27

**Authors:** Y. Bensalah, M. Sabir, F. Elomari

**Affiliations:** ^1^Psychiatric Hospital Ar Razi, Salé, Morocco

## Abstract

**Introduction:**

Several studies have demonstrated a high prevalence of sleep-related complaints in subjects with an addiction to psychoactive substances (alcohol, cannabis, nicotine, cocaine)

Sleep disorders negatively influence the quality of life of subjects suffering from addiction and increase the risk of relapse

**Objectives:**

To assess the prevalence of sleep disorders in patients with problematic use of psychoactive substances as well as associated factors

**Methods:**

This is a descriptive and analytical cross-sectional study carried out among 100 patients followed at Ar-Razi hospital Salé in Morocco for problematic use of psychoactive substances from June 1 to August 30, 2023

A questionnaire was used assessing the socio-demographic and clinical characteristics of our population

Sleep quality was assessed by the Pittsburgh Scale (PSQI)

**Results:**

There were 100 patients, with ages ranging from 18 to 56 years old and the majority of whom were males.

History of somatic pathology was reported in 36% of patients

The majority of patients had an associated anxiety disorder (60%)

The most consumed psychoactive substances were tobacco (95%), followed by cannabis, benzodiazepines and alcohol.

75% of patients reported poor sleep quality

There was a statistically significant difference between the risk of relapse and the reduction in sleep quality

**Conclusions:**

The prevalence of poor sleep quality in patients with disorders linked to psychoactive substance use is high, hence the importance of early detection in order to improve treatment.

**Disclosure of Interest:**

None Declared

